# Structure of Influenza A Virus Promoter and its Implications for Viral RNA Synthesis

**DOI:** 10.1100/tsw.2001.459

**Published:** 2001-12-04

**Authors:** Sung-Hun Bae, Byong-Seok Choi

**Affiliations:** Department of Chemistry, Korea Advanced Institute of Science and Technology, Taejon, Republic of Korea

**Keywords:** influenza A virus, RNA promoter, RNA dependent RNA polymerase (RdRp), viral RNA synthesis, transcription initiation, anti-viral drug

## Abstract

Since the worst worldwide pandemic ever recorded — the 1918 Spanish influenza outbreak that killed more than 20 million people — we have achieved significant advances in understanding the influenza virus. However, the fear of such a pandemic remains strong. For example, in 1997, when a lethal influenza variant afflicted eight people in Hong Kong, contributing to the death of six, officials feared the next wave had begun. They managed to solve the problem quickly, however, by destroying all of the poultry in Hong Kong[1].

Influenza is an RNA virus, the genome of which consists of eight single RNA strands, each coding one or two proteins. Using the host’s cellular machinery, influenza expresses ten proteins in total, including an RNA-dependent RNA polymerase (RdRp) complex made up of three proteins. The viral polymerase transcribes and replicates the viral genome but has no proofreading function, so the viral proteins are frequently mutated. Furthermore, combinatorial assembly of the genome segments can make many new variants when more than two different influenza strains infect the same host cell.

The 5' and 3' termini of each RNA genome segment are consistent among most influenza virus variants. They are recognized as promoters by the viral polymerase. The structure and function of this conserved RNA segment has been extensively studied by analyzing transcription or replication level according to specific mutations in the promoter, and also by modifying the solvent-accessible functional groups of this region[2,3,4,5]. A recent structural study of influenza A virus promoter shows that this conserved RNA region forms an overall A form helix containing significant bending ($46 \pm 10^{o}$) and has structurally unique and dynamic motifs in the asymmetric internal loop[6]. It reveals a structural basis for the specific interaction of influenza A promoter with the influenza virus polymerase.

In 1980, sequencing studies on influenza viruses revealed the presence of inverted complementary sequences in the 5' and 3' termini of each genome segment and suggested a RNA duplex structure so called a “panhandle”[7]. Its existence was demonstrated by using psoralen cross-linking[8], and several studies revealed that the panhandle is required in transcription initiation[2,3]. Despite the agreement on the importance of both the 5' and 3' ends, their functional structure has been debated. The “RNA-fork” model restricted the duplex region to the internal 6 base pairs and proposed a single strand structure for terminal 9 to 10 bases[13]. The "corkscrew" model agreed with the internal duplex region, but proposed a short-range intrastrand base pairing within each strand of the terminal region at the activated stage of viral RNA-polymerase interaction[4]. However, chemical modification and solution structural studies showed that 5' and 3' ends form an overall A form helix[5,9,10].

Recently it has been shown that both the 5' and 3' ends can bind the viral polymerase independently, but the binding affinity increases significantly when both the 5' and 3' ends exist[11]. Also, when the influenza A virus promoter was cloned into an internal position of a double-stranded RNA while the original promoter located in the terminal was mutated to disrupt base pairings, the initiation sites of the RNA synthesis were identical to those expected for the promoter in the internally displaced location[12].

From these results and our structural data[6], we infer that the influenza A virus promoter is a double-stranded helical structure. This structure has a possible regulatory role in the initiation of RNA synthesis, although it can be easily melted due to the inherent bending of the terminal stem and the solvent accessibility of the dynamic internal loop. The possible regulatory role might be related to the open complex formation in transcription initiation. A study on the abortive transcripts and the NTP requirement during the initiation of an influenza virus' transcription suggests that the open complex should extend to the +4 position, and the incorporation of AMP at the +5 position should induce the influenza RdRp to carry out transcriptional elongation[13]. Interestingly, this hypothesized boundary of the open complex coincides with the bending site at the +4 position of the terminal stem. Bending is known to lower the energy barrier to the melting of the double strands and to facilitate base pair opening in DNA.

This study provides new insight into the initial steps of RNA synthesis by the influenza A virus RdRp. The structural and dynamic features of the internal loop and the bending of the terminal stem would assist the viral RdRp to locate the starting site of RNA synthesis, and the bending property of the terminal stem may facilitate the melting of the RNA strands in open complex formation. Unlike the more well-known DdRp, the RdRp is for the most part virus-encoded polymerase, so its interaction with an RNA promoter is a promising target for highly specific anti-viral drugs. However, little has been deciphered about its promoter recognition and RNA synthesis mechanisms. More details on the structure of the RdRp and its interaction with RNA promoters are needed for a complete understanding of viral RNA synthesis and its application to anti-influenza drug development.
